# Effect of *N*-butyl cyanoacrylate on fracture healing in segmental rat tibia fracture model

**DOI:** 10.1186/s13018-014-0076-5

**Published:** 2014-09-11

**Authors:** Mehmet Akif Akcal, Oguz Poyanli, Koray Unay, Irfan Esenkaya, Bahadir Gokcen, Ayşe Sanem Fıratlıgil

**Affiliations:** Orthopaedics and Traumatology Clinic, Antalya Ataturk State Hospital, Antalya, 07040 Turkey; Orthopaedics and Traumatology Clinic, Goztepe Training and Research Hospital, Medeniyet University, Istanbul, 34722 Turkey; Istanbul Florence Nightingale Orthopedics and Traumatology Department of Spine Center, Istanbul, 34375 Turkey; Radiology Department, Balikligöl State Hospital, Urfa, 63000 Turkey

**Keywords:** Segmental fracture, *N*-butyl cyanoacrylate, Rat

## Abstract

**Background:**

Comminuted fractures can occur due to severe traumas. The treatment of these fractures that may cause serious morbidity and sometimes mortality is *N*-butyl cyanoacrylate. It has been reported that this adhesive provides sufficient rigid fixation for bone healing. This study aims to examine cyanoacrylate radiologically and histologically to determine whether it provides adequate recovery in segmental fractures. The secondary objective is to evaluate *N*-butyl cyanoacrylate, an adhesive material that can hold the fragments on the fracture line together following reduction.

**Methods:**

Sixteen Sprague–Dawley rats were divided in two groups as control (*n* = 8) and experimental (*n* = 8) groups. In the control group, segmental fractures were made and fixated with K-wire. In the experimental group, the same surgical procedure was applied and also fragments were stabilized with *N*-butyl cyanoacrylate.

**Results:**

On the sixth week, we did not see any statistically significant difference in the radiological scoring between groups. However, the pathological scores of the control group were statistically higher than the cyanoacrylate group.

**Conclusions:**

We found that cyanoacrylate was rapidly and easily applied in the segmental fractures but did not cause any superior radiological and clinical results compared to the control group. The cyanoacrylate had low viscosity, and it was not capable enough to fill the defects formed between osteotomy surfaces. However, it did not adversely affect fracture healing as seen in biopsies taken as a result of follow-ups.

## Introduction

Although numerous fixation methods have been devised to treat bone fractures, the search for optimal fixation methods continues. The method has to be minimally invasive, has to preserve soft tissues, blood supply, and trophics of the bone, and at the same time has to provide stable fixation [1]. *N*-butyl cyanoacrylate is reported in the literature that it is a polymeric, strong adhesive for the skin, soft tissues, and bones and that it does not cause a significant inflammatory response and does not compromise recovery [2,3]. Cyanoacrylate has become clinically and experimentally available in areas such as vascular surgery, plastic and reconstructive surgery, and ophthalmology [3-5].

Cyanoacrylate was synthesized by Airdiss in the year 1949, and its use in surgery as an adhesive was discovered in the year 1959 by Coover. By further modifications, a non-histotoxic form, butyl-2-cyanoacrylate, which had strong tissue-binding properties even in non-dry environments, was developed. Its use in fixation of fractures and osteotomies is still under investigation, but it has had promising results in the treatment of craniofacial and mandibular injuries [6]. *N*-butyl cyanoacrylate is approved by the American Food and Drug Administration as a tissue adhesive [7]. Also, Ahn et al. compared the biomechanical strength of pig calvarial segments after 8 weeks of fixation either with metal miniplates and biodegradable plates secured with *N*-butyl cyanoacrylate, and they found no significant difference in either histological assessment or biomechanical assessment of bone healing [8]. Esteves et al. analyzed the repair process of an autogenous bone graft in a block fixed with ethyl cyanoacrylate and 2-octyl-cyanocrylate adhesives in rat calvaria. They have stated that ethyl cyanoacrylate (ethyl group) and 2-octyl-cyanocrylate (octyl group) did not allow for graft incorporation, producing a localized and discrete inflammatory reaction which persisted at 60 days, being more intense in the octyl cyanoacrylate group [9]. Studies on butyl and ethyl cyanoacrylate have demonstrated an *in vivo* half-life of 24–48 weeks. It has been reported that the use of cyanoacrylate adhesives does not hinder the vascularization of newly formed bone [10].

It has been reported that cyanoacrylate can be used in the treatment of craniomaxillofacial fractures and osteochondral fractures of the talus as an alternative to plate screw and rigid fixation, and it has been demonstrated that it does not cause histotoxicity [1,5].

In the literature, the use of cyanoacrylate was generally described in the non-load-bearing cranio-facial skeleton; however, descriptions or comparative studies of the cyanoacrylate used in fracture research are still limited in detail.

Segmental fractures are frequently associated with serious, high-energy traumas [4]. In segmental fractures, the longitudinal blood circulation of the fragments that are in between is compromised due to the loss of connections at proximal and distal points and the severe soft tissue trauma added to that adversely influences circulation, thereby increasing the risk of inadequate fracture healing [7]. Our study aims to examine cyanoacrylate radiologically and histologically to determine whether it provides adequate recovery in segmental fractures. The secondary objective was to evaluate *N*-butyl cyanoacrylate, an adhesive material that can hold the fragments on the fracture line together following reduction in order to overcome the technical difficulty of keeping segmental fractures together, and we assessed its effects on fracture healing.

## Material and method

The ethics committee approval for this study was received. The rats were harbored individually in cages where they could access water and standard laboratory feed when they wanted in an environment at stable room temperature (20°C), where a rhythm of 12 h of light and 12 h of dark was applied. During the experiment, the rats were fed with tap water *ad libitum* and with standard rodent feed. No animals died until the end of the experiment.

### Surgical technique

**Figure 1:**
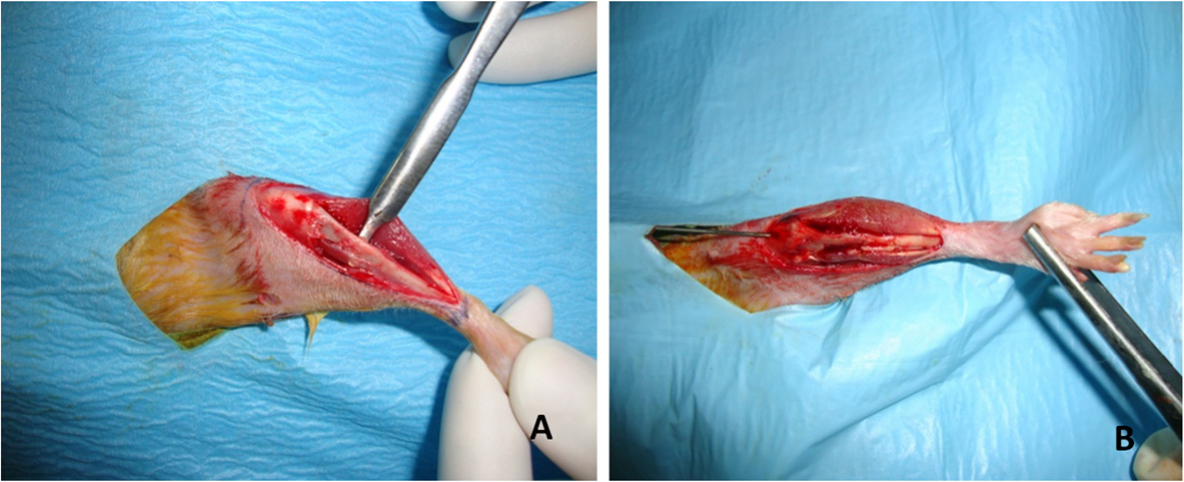
**Rat tibia. (A)** Preparation of the rat tibia for intervention. **(B)** Rat tibia with the segmental fracture formed and K-wire delivered is illustrated.

Anesthesia was achieved with the intra-muscular injection of ketamine (10 mg/kg). The left lower extremity of rats was shaved when they were in supine position, and then it was cleaned using a povidone-soaked sterile gauze. The lower extremities of subjects were brought to rotation and abduction positions to ensure superior migration of fibula; thus, the tibia between the knee joint and tarsal joint was palpated. Infra-patellar tibia was palpated and accessed via anteromedial incision. After the skin and cutaneous maximus muscle were dissected, the tibia was reached through the tibialis cranialis, peroneus longus, and brevis muscles. The tibial tuberositas caudal to the patellar joint was palpated and a 0.8-mm K-wire advanced until the tarsal joint was delivered. The three osteotomy lines were performed by manual force using 2-mm osteotome at nearly 10 mm caudal to the patellar joint and ending at 10 mm cranial to the tarsal joint to create segmental fractures in the tibial diaphysis area to create four different, equally spaced fragments (Figure 1).

### Trial protocol and grouping of test subjects

Sixteen adult male Sprague–Dawley rats included in the study were divided into two groups consisting of a control group (*n* = 8) and experimental group (*n* = 8). In both groups, an intramedullary fixation of the tibia was achieved initially using a 0.8-mm K-wire. No additional procedures were applied in the control group after the creation of segmental fractures as explained above. In the experimental group, two 0.1 g drops of *N*-butyl cyanoacrylate were applied on the anteromedial surface of the fracture line after the creation of the segmental fracture model. *N*-butyl cyanoacrylate was applied on the fracture lines using the special flow control apparatus of the manufacturing company with each drop being nearly 0.1 g. The fracture lines were kept in the appropriate anatomic position for 10 s, and then the skin was covered with 4–0 silk suture.

### Clinical and radiological evaluation

**Figure 2:**
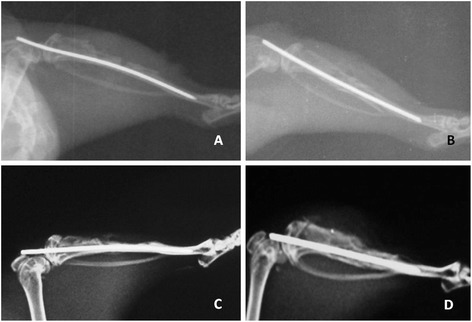
**Lateral X-rays of the control and the cyanoacrylate group on (A, B) day 1 and (C, D) week 6.**

Daily wound site care was applied in all subjects, and all subjects were daily assessed with respect to wound site infection and wound site dehiscence. Following the performance of surgical procedures on the subjects, conventional radiological studies were conducted four times on day 1, week 1, week 3, and week 6. The X-rays (Figure 2) were numerically scored based on the scoring system used by Akman et al. [11]. Briefly, no newly formed bone tissue or >2-mm bone defect was scored as 0 point, >1-mm defect was 1 point, <1-mm defect was 2 points, and 3 points were scored when the defect was not seen radiologically.

The rats in the control and study groups were followed up for 6 weeks; after which, they were sacrificed using high-dose ketamine anesthesia, and the K-wire in their left tibiae was removed. The entire tibia was removed by being detached from its surrounding soft tissue without harming the callus tissue. The fusion tissue in tibiae were subjectively evaluated by two persons who took part in the study independently from one another via macroscopic and biplanar examination. No movement on the fracture line (antero-posterior/lateral) was interpreted as full fusion (2 points), movement on one plane as secondary fusion (1 point), and movement on both planes as non-union (0 point) [11]. Radiological examinations were made in the control group and cyanoacrylate group in the antero-posterior and lateral directions on day 1, week 1, week 3, and week 6 following the surgical procedure. After the completion of radiological studies after a 6-week follow-up, the tibia was accessed via the incision scar line and the K-wire was withdrawn. The tibia was removed by being detached from the surrounding tissues without harming the callus formation. The fusion tissue in tibiae was subjectively evaluated by two persons who took part in the study independently from one another via macroscopic and biplanar examination.

### Histological assessment

After the completion of the conventional radiological study on week 6, the tibiae were fixated in 10% formol solution; the soft tissues were removed on the next day and transferred into 10% nitric acid. They were kept in this environment for 2 days and occasionally controlled by being taken out of the acid. One sample from each of the groups was prepared for decalcified histology. A single pathologist evaluated the specimens blindly. After the completion of decalcification procedure, the tissues were rinsed under running water for nearly 1 h to cleanse off the acid. A routine tissue follow-up procedure was performed. Sagittal sections of 5-μm thickness were cut using a microtome and stained with hematoxylin and eosin following the standard protocol. The stained cross sections enable the cellular structures at the fracture site to be visualized and allow identification of the various phases of the bone formation process present in each sample. They were examined under a light microscope. The callus tissue was scored based on the system recommended by Huo et al., and the scoring system would be seen in Table 1 [12].

**Table 1:** **The histological scoring system of Huo et al.** [1 **]**

**Score**	**Assessment of histology**
1	Fibrous tissue
2	Predominant fibrous tissue with minimal cartilage tissue
3	Cartilage tissue and fibrous tissue in a uniform manner
4	Predominant cartilage tissue with minimal fibrous tissue
5	Cartilage tissue
6	Predominant cartilage tissue with minimal immature bone
7	Immature bone and cartilage tissue in a uniform manner
8	Predominant immature bone with minimal cartilage tissue
9	Bone healing with immature bone
10	Bone healing with matured bone

### Statistical assessment

The statistical analyses in this study were completed using the NCSS 2007 software package. For the assessment of the data, descriptive statistical methods (median and quartile deviation) as well as Mann–Whitney *U* test for the comparison of dual groups, chi-square and Fisher's exact tests for the comparison of qualitative data, and McNemar's test for the repeated measurements of the qualitative data were used. In order to determine the consistency among observers, the weighted kappa (*κ*_w_) test was used. The results were assessed as follows: the significance was *p* < 0.05 and the confidence interval was 95%.

## Results

### Clinical and radiological findings

The subjects in the control and cyanoacrylate study groups were observed on a daily basis following the surgical procedure in terms of wound site infection and wound site dehiscence. In the control group, wound site dehiscence and wound site infection were observed in one of the eight subjects. In the cyanoacrylate study group, wound site dehiscence and wound site infection were observed in two of the eight subjects.

The averages of radiological scoring results obtained as a result of the assessments made by three independent researchers on day 1, week 1, week 3, and week 6. The average scores on week 6 are shown in Table 2. Radiological recovery was observed in neither the control group nor the study group during the radiological examination made on day 1 and week 1 following the surgical procedure. The researchers indicated that radiological recovery was present on weeks 3 and 6, and the assessment of the statistical consistency among three independent researchers (IRs) indicated that they were all consistent (*κ*_w_ = 0.698–0.784, *p* = 0.0001).

**Table 2:** **The average radiological scores were defined after surgical procedures were completed**

	**Control group**			**Cyanoacrylate group**		
	**IR 1**	**IR 2**	**IR 3**	**IR 1**	**IR 2**	**IR 3**
Day 1	0	0	0	0	0	0
Week 1	0	0	0	0	0	0
Week 3	2	2	3	2	1	2
Week 6	3	3	3	3	2	2

*IR* independent researcher. The scoring was made on a scale of 4. Score 0, no healing; score 1, callus formation; score 2, onset of osseous union; score 3, start of the disappearance of fracture line; score 4, complete osseous union.

The analysis of the radiological assessment is shown in Table 3. No statistically significant changes were observed in the radiological assessments of the control group on day 1, week 1, week 3, and week 6 (*p* = 0.293). No statistically significant changes were observed in the radiological assessments of the cyanoacrylate group on day 1, week 1, week 3, and week 6 (*p* = 0.018). No recovery was observed in all subjects in the control and cyanoacrylate groups on day 1 and week 1. On week 3, callus formation was identified in three subjects, and the onset of osseous union was identified in five subjects. In the cyanoacrylate group, callus formation was identified in three subjects, and the onset of osseous union was identified in five subjects. On week 3, it was observed that the radiological assessment distributions for the control group and cyanoacrylate group were the same (*p* = 1). On week 6, callus formation was identified in one subject, the onset of osseous union was identified in two subjects, and the disappearance of the fracture line was identified in five subjects. In the cyanoacrylate group, callus formation was identified in none of the subjects, the onset of osseous union was identified in three subjects, and the start of the disappearance of the fracture line was identified in five subjects. On week 6, no statistically significant difference was observed between the radiological assessment distributions for the control group and cyanoacrylate group (*p* = 0.549).

**Table 3:** **Statistical explanation of radiological scoring**

**Radiologic scoring**		**Control group**		**Cyanoacrylate group**		**Result**
		***n***	**%**	***n***	**%**	
Day 1	No recovery (0)	8	100.00	8	100.00	
Week 1	No recovery (0)	8	100.00	8	100.00	
Week 3	Callus formation (1)	3	37.50	3	37.50	*χ* ^2^ = 0, *p* = 1
	Onset of osseous union (2)	5	62.50	5	62.50	
Week 6	Callus formation (1)	1	12.50	0	0.00	*χ* ^2^ = 1.20, *p* = 0.549
	Onset of osseous union (2)	2	25.00	3	37.50	
	Disappearance of the fracture line (3)	5	62.50	5	62.50	

**Figure 3:**
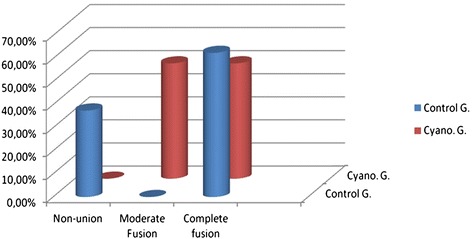
**Manual assessment of groups on week 6.**

The manual scoring results obtained are shown in Table 4. With respect to the manual assessment made by two IRs in both groups before the removal of the pathology on week 6, the manual assessments by the first IR and second IR were found to be statistically consistent (*κ*_w_ = 0.686, *p* = 0.0001). During the manual assessment, non-union was seen in three of the subjects, full fusion in five of the subjects, and secondary fusion in none of the subjects in the control group. In the cyanoacrylate group, secondary fusion was observed in four subjects, and full fusion was also observed in four subjects. No statistically significant difference was observed between the manual assessment distributions for the control group and cyanoacrylate group (*p* = 0.028, Figure 3).

**Table 4:** **Manual scoring of groups on week 6**

**Rat number**	**Control group**		**Cyanoacrylate group**	
	**Researcher 1**	**Researcher 2**	**Researcher 1**	**Researcher 2**
1	0	0	1	1
2	0	0	0	0
3	2	2	1	2
4	2	2	2	2
5	1	1	2	2
6	2	2	1	1
7	0	0	1	2
8	2	2	2	1

The scoring was made on a scale of 2. Score 0, non-union (movement present on both planes); score 1, secondary fusion (movement present on one plane); score 2, complete fusion (no movements).

### Histological findings

**Figure 4:**
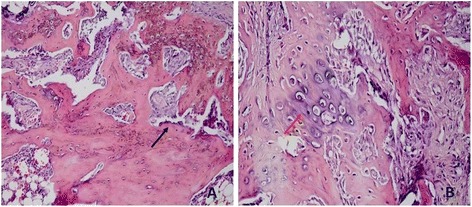
**Pathology scoring average. (A)** The pathology scoring average of the control group was 9, and the *black arrow* indicated the immature bone. **(B)** The pathology scoring average of the cyanoacrylate group was 8, and the *red arrow* indicated the cartilage tissue.

During the examination made under a light microscope using hematoxylin and eosin staining, no foreign body reactions, histotoxicities, inflammations, and cortical bone necroses were seen in the control group and the cyanoacrylate group (Figure 4). During the examination, the fracture lines were assessed for presence of fibrous tissue, cartilage tissue, and immature bone. The data were examined in accordance with the scoring system recommended by Huo et al. [13]. The scoring points obtained are shown in Table 5. Upon an assessment of the pathological scores of the groups, the pathological score averages of the control group were found to be statistically significant as compared to the cyanoacrylate group (*p* = 0.046). While recovery was observed with the immature bone in the control group, the presence of cartilage along with immature bone tissue was observed in the cyanoacrylate group (Table 6).

**Table 5:** **The scoring points obtained are shown in accordance with the scoring system recommended by Huo et al.** [13]

**Rat number**	**Control group**	**Cyanoacrylate group**
1	9	8
2	8	8
3	9	8
4	8	8
5	8	8
6	9	8
7	9	8
8	9	8

**Table 6:** **Assessment of pathological scores**

	**Control group**	**Cyanoacrylate group**	**MWU**	***p*** **value**
Pathological scoring	9 (8–9)	8 (8–8)	16	0.046

*MWU* Mann–Whitney *U* test.

## Discussion

Bone and tissue adhesives are common and beneficial supplements to standard methods of musculoskeletal tissue suture repair. Knowledge and development of biologically derived or inspired adhesives useful in orthopedic surgery are rapidly advancing. Recent literature demonstrates the increased adjunct or primary use of biological adhesives in the repair of musculoskeletal soft tissues, chondral fractures, and osteochondral fractures. The largest group of biologically derived adhesives and sealants is fibrin sealants, and other groups include gelatin-resorcin aldehydes, protein-aldehyde systems, collagen-based adhesives, polysaccharide-based adhesives, mussel adhesive proteins, and various biologically inspired or biomimetic glues [14].

The use of cyanoacrylate in the fixation of bone fragments is still being investigated. Gul et al. also used cyanoacrylate for the fixation of osteochondral fragments of the knee and achieved problem-free healing [15]. Furthermore, Yilmaz and Kuyurtar also reported in their study that cyanoacrylate was used successfully for the fixation of talar osteochondral fragments [6]. Amarante et al. reported in the results of their animal study assessing the healing and stability of fractured bone fragments that it was as effective as the use of metallic plates in the rigidity and healing of osteotomized cranial bone fragments [16]. Gonzalez et al. made an assessment in their study to see whether cyanoacrylate provided ease of use and rapid stabilization, and they reported that it provided ease of application, rapid stabilization, and good cosmetic results in the cranial bone flap model [17]. Toriumi et al. reported in their study where they investigated the toxicity of cyanoacrylate that butyl-2-cyanoacrylate had minimal toxic effect and that ethyl cyanoacrylate caused severe histotoxicity [5]. In our study, no foreign body reactions, histotoxicities, inflammations, and cortical bone necroses were seen in the control group and the cyanoacrylate group by the examination made under a light microscope using hematoxylin and eosin staining. In our study, no bone or soft tissue necroses and cortical bone resorptions were observed with *N*-butyl cyanoacrylate.

Even though *N*-butyl cyanoacrylate has no side effects on fracture healing, the pathological score averages of the control group were found to be statistically significant as compared to the cyanoacrylate group. While recovery was observed with the immature bone in the control group, the presence of cartilage along with immature bone tissue was observed in the cyanoacrylate group. However, there is no statistical significance between the control and the cyanoacrylate group in radiological and manual assessments. Whereas similar healing scores were obtained radiologically and manually in the cyanoacrylate group in our model of segmental fracture, stabilization of fracture lines was provided quickly and easily without damaging the surrounding tissues.

Our secondary objective was to evaluate *N*-butyl cyanoacrylate, an adhesive material that can hold the fragments on the fracture line together following reduction. It is suggested that keeping the bone fragments together for a long time using an adhesive agent may negatively impact fracture healing since this agent would create a mechanical barrier between the fracture lines. Therefore, the adhesive agent used should be of a temporary type and should not adversely affect fracture healing [13,18]. At a study conducted on dogs, isobutyl-2-cyanoacrylate was used for the fixation of osteochondral fragments, and it was shown that cyanoacrylate did not have a toxic effect on osseous tissue and did not adversely impact fracture healing. Furthermore, it was demonstrated that cyanoacrylate had low viscosity; hence, it was not capable enough to fill the defects formed between osteotomy surfaces on a rabbit model on account of its low viscosity [18]. The cyanoacrylate that we used in our study in order to enhance fixation in a segmental tibia fracture provides ease of application during use because of its low viscosity, and it was identified that it did not adversely affect fracture healing as seen in biopsies taken as a result of follow-ups. However, the promising result of our study was the pathological healing scores of the control and cynaoacrylate groups. Despite that the radiological and manual assessment scores were similar between the groups, recovery was observed with the immature bone in the control group. The presence of cartilage along with immature bone tissue was observed in the cyanoacrylate group; thus, pathological scores were lower in the cyanoacrylate group.

We propose that low viscosity of cyanoacrylate provides rapid fixation of fragments and also does not allow it to infiltrate through the fracture line. The first clue of this proposal is that no foreign body reactions, histotoxicities, inflammations, and cortical bone necroses were seen in the cyanoacrylate group. The limitation of our study is that it does not explore and illustrate the histology and also the reaction of regenerative and inflammatory cells in direct contact to cyanoacrylate.

In the literature, the use of cyanoacrylate was generally described in the non-load-bearing cranio-facial skeleton; however, there are no experimental studies related to its use in load-bearing segmental fractures. The studies pertaining to the successful use of cyanoacrylate in the fixation of talar osteochondral fragments and the use of cyanoacrylate in a case for the fixation of osteochondral fragments of the knee are case presentations [2,7,16]. The necessary experimental studies should be completed before cyanoacrylate can start to be commonly used in clinical practice. The histotoxicity and bioavailability experiments are adequate. In this study, we aimed to show that the treatment of segmental tibial bone fractures may be easily and reliably treated with *N*-2-butyl cyanoacrylate. We found that cyanoacrylate was rapidly and easily applied in the segmental fractures, but it did not cause any superior radiological and clinical results compared to the control group. However, the use of *N*-butyl cyanoacrylate did not affect bone healing negatively and did not cause cortical bone resorption.
